# Confronting the Upstream Causes of COVID-19 and Other Epidemics to Follow

**DOI:** 10.1177/0020731420946612

**Published:** 2020-08-03

**Authors:** Howard Waitzkin

**Affiliations:** 1 Department of Sociology and Health Sciences Center, University of New Mexico, Albuquerque, New Mexico, USA

**Keywords:** agriculture, capitalism, pandemic, upstream causes

## Abstract

The upstream causes of the COVID-19 pandemic have received little attention so far in public health and clinical medicine, as opposed to the downstream effects of mass morbidity and mortality. To resolve this pandemic and to prevent even more severe future pandemics, a focus on upstream causation is essential. Convincing evidence shows that this and every other important viral epidemic emerging in the recent past and predictably into the future comes from the same upstream causes: capitalist agriculture, its destruction of natural habitat, and the industrial production of meat. International and national health organizations have obscured the upstream causes of emerging viral epidemics. These organizations have suffered cutbacks in public funding but have received increased support from international financial institutions and private philanthropies that emphasize the downstream effects rather than upstream causes of infectious diseases. Conflicts of interest also have impacted public health policies. A worldwide shift has begun toward peasant agricultural practices: Research so far has shown that peasant agriculture is safer and more efficient than capitalist industrial agricultural practices. Without such a transformation of agriculture, even more devastating pandemics will result from the same upstream causes.

While struggling to lessen the many downstream effects of COVID-19, through dangerous and sometimes heroic efforts, we in medicine and public health also must try to maintain a clear and relentless focus upstream on the root causes of the pandemic. Clarity about the upstream causes rarely emerges in current discussions of the pandemic, even in the scientific and public health communities, and hardly ever in pronouncements of the public health institutions on which many of us rely: the World Health Organization (WHO), U.S. Centers for Disease Control and Prevention (CDC), U.S. National Institute of Allergy and Infectious Diseases, Pan American Health Organization, Gates Foundation, and so forth. Identifying the upstream causes of epidemics has been a central goal of epidemiology since the 1840s, when the pathologist Rudolf Virchow did his path-breaking investigation of the typhus epidemic in Upper Silesia. Due to the current pandemic’s magnitude, one would expect that the upstream causes would be crystal-clear for all to see, so we could address them directly, but amazingly this is not the case.

Many people attribute the origins of the pandemic to the strange and retro marketing practices of some individuals and groups in Wuhan, China, who were selling wild animals in the market from which the virus spread, eventually worldwide. But such marketing practices have been going on for a long time, probably hundreds of years or more. Why do we have a pandemic now and not earlier?

## Upstream Causes in Agriculture

This pandemic and every other important emerging viral epidemic in the recent past and predictably into the future come from the same upstream causes: capitalist industrial agriculture, destruction of natural habitat, and production of meat. In recent decades, the intensity and worldwide scale of these practices have increased rapidly. Pioneering microbiological and epidemiological studies have clarified these upstream causes of emerging epidemics, whose effects we now are confronting every day.^1–6^ In addition to viral epidemics, these and similar agricultural practices also deepen the parallel crises of multi-drug-resistant bacterial infections (through overuse of antibiotics in industrial meat and fish production), climate change (by destruction of rainforest habitats and long-distance transportation of food that requires burning of petroleum), plastic pollution (by agricultural packaging methods), and other severe environmental problems.

Natural forest habitat previously provided ecological control for microbes such as SARS-CoV-2 and their hosts, such as bats. Clearing habitat for industrial agriculture emerged as a central characteristic of China’s economy as it “liberalized” after Mao Zedong into a bastion of the capitalist world system.^7^ Similar zoonotic sources of transmission from destroyed habitats have happened in China with the previous coronavirus in severe acute respiratory syndrome (SARS); Ebola in Africa; Zika in Africa, Latin America, and elsewhere; and arguably HIV in Africa.^8–10^

Another practice stemming from the capitalist model of agriculture involves industrial production of meat. Especially for pigs and chickens but also other species, reproduction of offspring, growth to adulthood, slaughter, and packaging increasingly occur under factory conditions that receive little regulatory oversight and control. Worldwide, a small number of large, oligopolistic, multinational corporations dominate factory farming. As a result, viral contamination and mutations to more virulent organisms in unsanitary factory conditions have led to epidemics of swine flu, avian flu, and a variety of emerging influenza viruses.^2–5^

## Deemphasis on Agriculture in Public Health

Do sources like WHO, CDC, and the Gates Foundation provide a complete picture? Some well-motivated people work for these agencies, and much helpful information is available. But mistakes get made, as have occurred multiple times during the COVID-19 pandemic, and more importantly these sources rarely address the upstream causes of epidemics. Many have commented about the devastating funding cutbacks and de-prioritization that have crippled these organizations’ capacity to protect public health. As just one example, the annual program budget of WHO for the whole world is smaller by about half than the operating budget of a large medical center in the United States (WHO: $4.34 billion; New York Presbyterian Hospital: about $8 billion).^11,12^

Into the financial crisis of international health institutions have stepped the World Bank, International Monetary Fund, Gates Foundation, and other agencies of “philanthrocapitalism,” whose financial priorities and ideologies dominate the policies and practices of WHO and its affiliated organizations worldwide.^13^ This is one reason that the global People’s Health Movement produces “WHO Watch” and “Global Health Watch” to monitor WHO critically and to offer alternatives that WHO and its affiliates do not pursue because of their financial dependency on international financial institutions and philanthrocapitalism.^14^ Partly due to such financial support, international and national health organizations almost always promote reductionist initiatives that focus on so-called magic bullets such as vaccines and antiviral medications, as well as behavioral change at the level of individuals, rather than upstream causes.

Financial conflicts of interest also can distort the organizations’ policies. For instance, the Gates Foundation has invested in and promoted genetically modified crops through such corporations as Monsanto/Bayer. Farmlands for such crops lead largely to the production of animal feeds, required for increased meat production, which causes further loss of forest habitat.^15^ In addition, Gates’ investments emphasize pharmaceutical corporations and other companies that profit from intellectual property, which in the realm of computer software creates most of Gates’ wealth. Similarly, CDC and its employees regularly attract criticism based on revelations about conflicts of interest at both the organizational level (especially regarding grants and other financial support that a foundation connected to CDC receives from the pharmaceutical industry) and individual level (employees’ and committee members’ investments in and gifts from industry).^16^

## Agricultural Corporations

So WHO, CDC, Gates, and their affiliates have obscured the upstream causes of emerging viral epidemics not only in COVID-19 but also in all other recent epidemics. An especially disheartening example (I was involved) was the swine flu epidemic of 2009, which began within 1 mile of Smithfield Foods’ notorious industrial pig farm operation in a rural area of Veracruz state in Mexico. Smithfield Foods had outsourced this operation from the United States partly to avoid occupational and environmental cleanup requirements. Although Mexican public health authorities and investigators reported this epidemiological association between swine flu and capitalist industrial agriculture, CDC, WHO, and all other international health organizations pursued reductionist strategies such as a vaccine, rather than confront radical change in the meat processing industry.

During the COVID-19 pandemic, Smithfield’s practices became even more startling. Less than a decade after the swine flu epidemic, a Hong Kong-based investment corporation, WH Group Ltd, had acquired Smithfield Foods. In 2018 Smithfield executives based at U.S. headquarters in Smithfield, Virginia, welcomed the ongoing epidemic of African swine fever because a reduced global supply of pork would lead to major increases in prices and profitability for the corporation.^17^ When COVID-19 struck, Smithfield executives reassured U.S. consumers that, despite ownership in Hong Kong, the corporation did not import pork from China but instead exported U.S. pork to China, where prices were higher.^18^ A Smithfield pork processing plant in Sioux Falls, South Dakota, became one of the largest COVID-19 hotspots in the United States. Rapid spread of the infection to workers because of similarly unsanitary working conditions threatened to close other meat-producing plants as well.^19^

The role of capitalist industrial agriculture through loss of habitat and meat production occasionally does surface in the mainstream media. Such media attention, while limited, happened recently regarding the sources of COVID-19, although the term “capitalist” did not enter the discussion.^20^ But the impacts of such corporations on emerging epidemics rarely appear in communications or policies of international health organizations or the Gates Foundation.

Leaders of these agencies are fully aware that emerging viral epidemics come from capitalist industrial agriculture. They showed this awareness in Event 201 on October 18, 2019, ironically about 2 months before the COVID-19 epidemic began in Wuhan.^21^ In this “tabletop exercise,” coordinated by the Johns Hopkins Center for Health Security, Gates Foundation, and World Economic Forum, a novel coronavirus pandemic begins at pig farms in Brazil and spreads rapidly around the world, resulting in 65 million deaths and catastrophic effects on the global economy, political stability, and international security. After the COVID-19 epidemic actually began, the sponsors of Event 201 emphasized that they did not predict the timing of COVID-19 and that the projected death toll did not necessarily apply. But they did not say anything about an initiative to eradicate the practices of capitalist industrial agriculture that led to the hypothetical scenario of Event 201, to the current global COVID-19 pandemic, and to the inevitable future pandemics that will occur on a similar scale or even worse.

## Better Ways to Produce Food

Is there an alternative to capitalist industrial agriculture? Yes. Around the world, often against resistance from corporations and governments, farmers are returning to peasant agricultural practices. A whole body of research has shown that peasant agriculture is not only safer than capitalist agriculture but actually is more efficient and productive as well.^22^ Millions of people worldwide already are making this transition, often because they/we see no other choice. Especially in the context of economic collapse, capitalist agriculture – with its tendency to overproduce and even destroy surplus food while hunger and food insecurity worsen – is ill suited to feed the world’s peoples.

Changing the upstream causes of epidemics such as COVID-19 and others yet to come becomes a key scientific and practical priority for medicine and public health, considering the future of humanity and other inhabitants of the planet. If that transformation doesn’t happen, we can expect even more devastating pandemics, stemming from the same upstream causes.
